# Entering and Exiting the Vehicle: Day-to-Day Activity, or High-Risk Endeavor Among Older Adults

**DOI:** 10.1093/geroni/igaf032

**Published:** 2025-03-19

**Authors:** Xingyu Zhang, Yang Wang, Julie Faieta

**Affiliations:** Department of Communication Science and Disorders, University of Pittsburgh, Pittsburgh, Pennsylvania, USA; School of Computing and Information, University of Pittsburgh, Pittsburgh, Pennsylvania, USA; Department of Rehabilitation Science and Technology, University of Pittsburgh, Pittsburgh, Pennsylvania, USA

**Keywords:** Community mobility, Fall risk, Transportation

## Abstract

**Background and Objectives:**

A primary concern of many older adults as they age is whether they will be able to remain in the community setting. This is impacted by their ability to commute to their surroundings as needed to attend occupational-, leisure-, or health care-related appointments in the community setting. Whether an older adult is able to independently drive or relies on rides from others, the first step to safe community transportation is boarding into and alighting out of a vehicle. This study aimed to determine the prevalence of boarding and alighting injuries across age demographics and describe demographic factors that may impact the prevalence of injury.

**Research Design and Methods:**

This study was a retrospective, cross-sectional analysis. National Electronic Injury Surveillance System-All Injury Program (NEISS-AIP) data from 2017 to 2021 was analyzed through descriptive statistics and multivariable logistic regression.

**Results:**

Findings indicate that the annualized estimate of injury is 209,797 across age groups. Older adults were found to have a higher rate of injury and hospitalization following injury.

**Discussion and Implications:**

This study replicates earlier reporting on non-crash vehicle injuries occurring during boarding and alighting from 2001 to 2003. Continued research is needed to investigate methods of boarding and alighting injury risk reduction for vulnerable populations.

**Translational Significance:** This study addresses the lack of up-to-date insight into the prevalence of risk factors associated with vehicle boarding and alighting injuries. Findings indicate a higher prevalence of boarding and alighting injuries among older adults and higher rates of hospitalizations for older adults who are injured during boarding and alighting tasks. This article provides current real-world prevalence and demographic data that can be utilized to drive injury prevention efforts among vulnerable, aging groups.

The aging global population provides the opportunity to support health span for community dwelling older adults. Advances in smart home technology continue to work toward sustained function and safety in the home context (Moyle et al., 2021; Pietrzak et al., 2014). Sustained ability to reside safely in the home is critically important as 77% of older adults in the United States express a desire to stay in the home setting as they age (AARP, 2021). However, aging well for many is not limited to the home context but rather includes home and community access. Community access allows for engagement in meaningful occupations, social, and leisure activities, which can, in turn, facilitate health and satisfaction among older adults (Michèle et al., 2019). For most individuals in the United States, community access requires either community or personal vehicle transportation. Whether riding or driving a vehicle, the initial and final access point is vehicle entry and exit, which we will refer to here as boarding and alighting. Boarding and alighting are more complex than most sit-to-stand transfers as there is leaning, ducking, and twisting involved. Therefore, it is reasonable to postulate that boarding and alighting from a vehicle can become increasingly difficult and hazardous with age-related decline in strength and mobility. Anecdotally, many have, and continue to, assist aging family members and friends into and out of vehicles to ensure their safety. Rehabilitation professionals, namely occupational therapists, can provide guidance on safe methods of assisting at-risk individuals into or out of a vehicle and on how someone who is still independent can safely self-board and alight from a vehicle. There is limited literature informing on the prevalence and impact of boarding and alighting risk and injuries. Such information is needed to validate whether additional attention should be given to supporting this aspect of access.

## Potential Impact of Boarding and Alighting Risks

The risk of fall injury during vehicle boarding and alighting is of particular concern for older adults who experience age-related decline in strength, mobility, and balance. The consequences of falls among community-dwelling older adults have been summarized according to the percentage affected. King and Tinetti (1995) summarize the literature to report that 4%–6% sustain a fracture, 39%–50% are unable to get up after they experience a fall, and 41%–43% have decreased activity following their falls. These outcomes can have dire consequences on the quality of life and ability to age well and independently for this demographic. In their Clinical Practice Piece on fall prevention, Ganz and Latham (2020) note that “falls often result from interacting risks that can be reduced or managed,” describing both intrinsic person-related risks, and then extrinsic interaction with one’s environment. This then prompts the question—of the 14 million falls occurring in the US annually, how many can be prevented by improving the safety in vehicle boarding and alighting (U.S. Centers for Disease Control and Prevention, 2024)? Furthermore, how do we approach the risks, whether person-, activity-, or context-related that people encounter in routine vehicle engagement?

### Study Model: Person-Environment-Occupation

The Person-Environment-Occupation (PEO) Model is an approach used to conventionalize “the person, his/her environments and occupations dynamically interacting over time” (Strong et al., 1999). In other words, the PEO model considers the occupations that an individual finds meaningful and looks at how the individual can be successful in these tasks in their individual environments (Law et al., 1996). With this frame of reference, we anticipated that the greater the overlap between the person and their success in meaningful occupations within the context of their environment, the greater their functional satisfaction in day-to-day life. The inability to safely access a vehicle can effectively impede their access to the community and the professional, social, and leisure activities that occur outside of the home. The environment. with respect to vehicle access and use, should consider the general practices and values with respect to community transportation—some countries, states/provinces, or cities are very conducive to community transportation (e.g., bus, train, metro, etc.) or ambulatory commutes. Other environments tend toward individual vehicle ownership and autonomy in commuting across broader areas (e.g., suburb residents commuting to jobs in a neighboring city or city residents commuting to jobs in neighboring towns). We must consider the occupation of riding or driving, the contextual norms of autonomy in vehicle access, and the subsequent occupations that are negatively impacted when one cannot safely board and alight from a vehicle.

Dellinger, Boyd, and Haileyesus (2008) gathered the National Electronic Injury Surveillance System-All Injury Program (NEISS-AIP) data from 2001 to 2003 and assessed the annualized boarding and alighting injury rates among older adults from within that time period. They report a rate of 37,000 boarding and alighting injuries that result in emergency room department visits yearly. This statistic begs additional attention to this matter as we work to support a quickly aging population that is, by and large, seeking to remain in the home. Our study seeks to replicate their findings in a more recent and larger sample through the NEISS-AIP database in order to: (a) determine the present prevalence of boarding and alighting injuries; and to (b) describe demographic variables associated with increased prevalence.

## Method

### Data Source

The NEISS-AIP is a database managed by the U.S. Consumer Product Safety Commission (CPSC) that collects data on all types and causes of nonfatal injuries treated in hospital emergency departments (EDs) in the United States. NEISS-AIP provides a nationally representative sample of data from approximately 66 hospitals selected based on hospital size and geographic location to ensure broad coverage reflective of the U.S. population. This system includes hospitals that have a 24-hour ED and a minimum of six beds. Data are gathered from patient medical records during each ED visit involving an injury, detailing the injury mechanism, diagnosis, demographics, and ED visit outcomes (such as whether the patient was treated and released or hospitalized).

Each injury case recorded in the NEISS-AIP has an assigned sample weight inversely proportional to the hospital’s selection probability, allowing researchers to generate national estimates from the sample data. This weighting ensures that the findings represent the broader national context regarding injury rates and patterns, despite the data originating from a relatively small number of hospitals. The confidentiality of patient information is strictly maintained, with data used solely for statistical purposes aimed at improving consumer safety and preventing injuries.

### Study Design

This study centered on vehicle boarding and alighting-from-vehicle injuries and analyzed data from a specific period, covering the years 2017 to 2021. All persons who were unintentionally injured while boarding or alighting from various types of motor vehicles, such as cars, trucks, vans, buses, and sport utility vehicles were included. Incidents may have occurred in a range of locations, including on or off streets and highways, as well as in driveways and parking lots. Injuries sustained from striking against or falling off the exterior of a motor vehicle or the back of an open bed of a pickup truck were excluded, as these were not related to the primary focus of entering or exiting a vehicle.

### Statistical Methods

Descriptive statistical methods were utilized to delineate the distribution of injuries across various age groups. Injury rates per 100,000 population were computed using weighted estimates (weights are as reported in the data set) to elucidate the relative frequency of injuries across diverse age demographics. In addressing the complexities of the sampling design, the design effect was computed to ensure that standard errors and confidence intervals faithfully represented the sampling strategy. For the analysis of rate stability and statistical significance across different demographic and situational subgroups, the Kish formula was implemented for variance estimation of rate ratios. This formula is exceptionally well-suited to survey data characterized by complex sample designs, providing a robust framework for the variance estimation of derived ratios.

We stratified the annualized injury counts and further segmented the estimates by years and demographic groups. This stratification allowed for an extended analysis incorporating detailed demographic breakdowns, to accurately identify high-risk groups. Continuing with the rate analysis, we calculated injury rates per 100,000 population, each complemented by 95% confidence intervals. The key covariates such as sex, race/ethnicity, affected body area, primary diagnosis, and patient disposition post-incident were evaluated to discern patterns in injury rates and outcomes.

A multivariable logistic regression was conducted to explore the relationships between various predictors and the likelihood of hospitalization following an injury. Odds ratios were estimated to gauge the strength of these associations, with corresponding 95% confidence intervals providing a spectrum of plausible values. Significance levels were set at 0.05, and all data analyses were performed using SAS Version 9.4.

## Results

From the data spanning 2017 to 2021, a total of 17,431 sample cases were documented, leading to an estimate of over 1,086,473 individuals sustaining injuries during the boarding and alighting process. The annual estimate of injuries across all age groups was approximately 217,295. Notably, adults aged 65 and older accounted for 28.12% of the total injuries, a significant increase from the 12% reported in the 2001–2003 statistics. The injury rate per 100,000 persons was 54.51 for those under 65, but this rate increased to 107.6 for those aged 65 and above, marking a notable change compared to the results from 2001 to 2003 (109.2 for those under 65 and 104.5 for those 65 and older). Further analysis of the older age groups revealed that injury rates increased with age as can be seen from Table 1. For instance, the rate for the 75–84 age group was 131.8, whereas it escalated to 199.2 for those aged 85 and older.

**Table 1.** All Boarding and Alighting Injuries According to Age Group: United States, 2017–2021

Age	Sample size, *n*	Weighted estimate, *n*	Weighted percentage (%)	Annualized estimate, *n*	Rate per 100,000 population	95% CI for rate
<65	13,252	754,039.20	71.88	150,807.84	54.51	53.6–55.4
≥65	4,179	294,946.50	28.12	58,989.30	107.59	104.7–110.4
65–74	1,810	125,161.20	11.93	25,032.24	77.33	85.1–79.2
75–84	1,444	105,216.20	10.03	21,043.24	131.75	127.2–136.3
≥85	925	64,569.07	6.16	12,913.81	199.19	193.6–204.7
Unknown	822	37,486.93				
All age	17,431	1,086,472.63	100.00	217,294.53	65.55	64.7–66.5

*Note*: CI = confidence interval.

Table 2 categorizes the injuries related to boarding and alighting from different dimensions such as age groups, gender, injured body parts, diagnoses, and dispositions. Women accounted for a higher proportion of injuries (55.1%) compared with men (44.9%). When considering different age groups, the gender disparity is more pronounced in the age group of 65 and older, with a difference of 18.8% between men and women, compared with a 7% difference in the under-65 age group. Regarding the primary body area affected, adults aged 65 and older had a significantly higher probability of head/neck injuries (31.1%) compared with adults under 65 (19.7%). Additionally, the likelihood of lower trunk injuries in adults aged 65 and older (15.3%) was nearly twice that of younger adults (8.1%). However, for the arm/hand, younger adults (under 65) had a much higher injury rate (32.2%) than older adults (14.9%). We also found that fractures were the most common diagnosis among adults 65 and older (26.9%), while younger adults were more prone to strains and sprains (24.8%). The hospitalization rate for older adults (21.3%) was approximately five times higher than that for younger individuals (3.9%).

**Table 2.** Boarding and Alighting Injury: Annualized Weighted Estimates, United States

Characteristic	Age <65		Age ≥65		All Age	
	Annualized estimate, *n*	Weighted percentage	Annualized estimate, *n*	Weighted percentage	Annualized estimate, *n*	Weighted percentage
Overall	150,808		58,989		209,797	
Sex						
Female	80,669	53.5	35,029	59.4	115,698	55.1
Male	70,133	46.5	23,961	40.6	94,094	44.9
Race/ethnicity						
White	74,828	49.6	30,597	51.9	105,425	50.3
Black	21,918	14.5	3,824	6.5	25,742	12.3
Other	54,061	35.8	24,569	41.6	78,630	37.5
Primary body area affected						
Head/neck	29,679	19.7	18,360	31.1	48,039	22.9
Upper trunk	8,155	5.4	4,692	8.0	12,847	6.1
Lower trunk	12,149	8.1	9,016	15.3	21,165	10.1
Arm/hand	51,834	34.4	8,787	14.9	60,622	28.9
Leg/foot	48,486	32.2	17,917	30.4	66,403	31.7
Other or unknow	505	0.3	217	0.4	722	0.3
Primary diagnosis						
Contusions, abrasions	34,243	22.7	10,650	18.1	44,893	21.4
Laceration	15,609	10.4	8,448	14.3	24,057	11.5
Fracture	22,809	15.1	15,866	26.9	38,676	18.4
Strain/sprain	37,380	24.8	6,386	10.8	43,766	20.9
All other	40,767	27.0	17,639	29.9	58,406	27.8
Disposition						
Hospitalized	5,868	3.9	12,591	21.3	18,459	8.8
Other	144,940	96.1	46,398	78.7	191,338	91.2

Across all age groups, the injury rate for boarding and alighting is higher in women (69.3) compared to men (57.2), with a greater gender disparity observed in the over-65 age group in which case the rate was higher for men (women at 97.6 and men at 115.7). Table 3 indicates that, except for the arm/hand where no significant differences were noted, the injury rates for all other body parts are significantly higher in the over-65 age group compared to those under 65. The rates of fractures, contusions, abrasions, and lacerations are also higher in the older age group, while the rates of strains and sprains show no difference between age groups. The hospitalization rate for older adults is more than 10 times higher than that for those under 65, a proportion that is consistent with the findings from the 2001 to 2003 data.

**Table 3.** Boarding and Alighting Injury: Annualized Injury Rates Per 100,000, United States

Characteristic	Age <65			Age ≥65			All Age		
	Rate	95% CI		Rate	95% CI		Rate	95% CI	
		LL	UL		LL	UL		LL	UL
Sex									
Female	59.0	58.1	59.9	115.7	113.1	118.3	69.3	68.6	70.0
Male	50.1	49.5	50.7	97.6	94.3	100.9	57.2	56.6	57.8
Race/ethnicity									
White	36.4	34.4	38.4	66.7	63.4	70.0	41.9	39.3	44.5
Black	55.3	51.5	59.1	72.1	66.0	78.2	57.3	53.2	61.4
Other	172.7	161.3	184.1	671.2	600.4	742.0	224.9	205.8	244.0
Primary body area affected									
Head/neck	10.7	10.5	10.9	33.5	32.5	34.5	14.5	14.3	14.7
Upper trunk	2.9	2.8	3.0	8.6	8.3	8.9	3.9	3.8	4.0
Lower trunk	4.4	4.3	4.5	16.4	15.7	17.1	6.4	6.2	6.6
Arm/hand	18.7	18.3	19.1	16.0	15.6	16.4	18.3	17.9	18.7
Leg/foot	17.5	17.2	17.8	32.7	31.8	33.6	20.0	19.8	20.2
Other or unknown	0.2	0.2	0.2	0.4	0.3	0.5	0.2	0.2	0.2
Primary diagnosis									
Contusions, abrasions	12.4	12.2	12.6	19.4	18.7	20.1	13.5	13.3	13.7
Laceration	5.6	5.5	5.7	15.4	14.9	15.9	7.3	7.2	7.4
Fracture	8.2	8.0	8.4	28.9	28.1	29.7	11.7	11.5	11.9
Strain/sprain	13.5	13.3	13.7	11.6	11.1	12.1	13.2	13.0	13.4
All other	14.7	14.4	15.0	32.2	31.3	33.1	17.6	17.3	17.9
Disposition									
Hospitalized	2.1	1.9	2.3	23.0	22.0	24.0	5.6	5.4	5.8
Other	52.4	51.9	52.9	84.6	82.5	86.7	57.7	57.0	58.4

*Note*: CI = confidence interval; LL = lower limit; UL = upper limit.

Table 4 provides a multivariable logistic regression analysis to examine factors associated with the need for hospitalization. Adults aged 65 and older, including younger-older adults (65–74), middle-older adults (75–84), and oldest-older adults (≥85) have a higher likelihood of hospitalization compared with adults under 65, with an odds ratio of 2.93 (95% CI: 32.48–3.46), 4.26 (95% CI: 3.59–5.06), and 6.18 (95% CI: 5.09–7.51), respectively. This indicates that age is a significant factor influencing hospitalization. Men, compared with women, have a 39% increased chance of hospitalization (odds ratio of 1.39, 95% CI: 1.23–1.57), suggesting that gender is also a determinant of hospitalization needs. Compared with White adults, adults of other races have a higher risk of hospitalization, whereas the hospitalization risk for Black adults does not significantly differ from that of White adults. Regarding the affected body area, patients with lower trunk injuries are more likely to be hospitalized (odds ratio of 1.92, 95% CI: 1.56–2.35), whereas patients with injuries to the arm/hand are significantly less likely to require hospitalization (odds ratio of 0.10, 95% CI: 0.08–0.13). Compared with other types of injuries, patients with fractures have a significantly higher likelihood of hospitalization (odds ratio of 12.80, 95% CI: 10.26–15.96). The likelihood of hospitalization for strains or sprains is significantly reduced (odds ratio of 0.25, 95% CI: 0.17–0.37).

**Table 4.** Multivariable Logistic Regression for Hospitalization

Variable	Model from unweighted data				Model from weighted data			
	OR	95% CI		*p*	OR	95% CI		*p*
		LL	UL			LL	UL	
Age								
<65 (reference)								
65–74	2.93	2.48	3.46	<.0001	3.04	2.48	3.72	<.0001
75–84	4.26	3.59	5.06	<.0001	4.08	3.32	5.01	<.0001
≥85	6.18	5.09	7.51	<.0001	6.03	4.79	7.61	<.0001
Sex								
Female (reference)								
Male	1.42	1.25	1.61	<.0001	1.50	1.29	1.75	<.0001
Race								
White (reference)								
Black	1.15	0.94	1.41	.1659	1.38	1.07	1.76	.0117
Other	1.65	1.44	1.89	<.0001	1.77	1.51	2.08	<.0001
Primary body area affected								
Head/neck (reference)								
Upper trunk	0.66	0.50	0.86	.0023	0.66	0.48	0.93	.0172
Lower trunk	1.90	1.55	2.34	<.0001	1.71	1.36	2.17	<.0001
Arm/hand	0.10	0.08	0.13	<.0001	0.12	0.09	0.17	<.0001
Leg/foot	0.66	0.55	0.79	<.0001	0.70	0.56	0.87	.0014
Other or unknown	3.27	1.72	6.22	.0003	3.42	1.70	6.90	.0006
Primary diagnosis								
Contusions, abrasions (reference)								
Laceration	0.65	0.46	0.93	.0176	0.78	0.52	1.16	.2204
Fracture	12.92	10.35	16.12	<.0001	12.16	9.30	15.88	<.0001
Strain/sprain	0.26	0.17	0.39	<.0001	0.25	0.15	0.40	<.0001
All other	2.57	2.04	3.23	<.0001	2.35	1.79	3.08	<.0001

*Note*: CI = confidence interval; LL = lower limit; UL = upper limit.

## Discussion

The goal of this study was to determine the present prevalence of boarding and alighting injuries and to describe demographic variables associated with increased prevalence. To do this, we investigated publicly available NEISS-AIP data spanning a four-year period (2017–2021). This NEISS-AIP data for various time periods has been used extensively throughout the injury literature, particularly to explore injuries among older adults and to explore fall injury data (Navon et al., 2023; Office of Disease Prevention and Health Promotion, n.d.). We modeled our analyses after those reported by Dellinger, Boyd, and Haileyesus (2008), who reported on a 2001–2003 time period, in order to evaluate variation in boarding and alighting injuries across time (Dellinger et al., 2008). The rate of injury decreased for individuals <65 years of age but increased for younger-older adults (65–74), middle-older adults (75–84), and oldest-older adults (≥85). The causes behind a change in the rate of injury are difficult to posit since the data set does not provide medical notes to describe the manner of injury, nor does it provide the presence or absence of comorbid conditions that could provide insight into the profile of those who are sustaining injury. Since the initial investigation (2001–2003) numerous vehicle safety technologies have been developed, become more common, or have even been made mandatory by law (e.g., back-up cameras, parking, and lane assist, hands-free phone technology, and built-in GPS [2008]). Whether vehicle design changes, technological advances, or other external factors have influenced the reduced rate of falls in younger adults or the increased rate of falls in older adults remains to be seen.

As noted, the PEO model guides a multifaceted perspective that considers the person (those at risk or affected), the occupation (boarding and alighting from vehicles as an early step in driving or riding tasks), and the context (the rider/driver environmental and societal context). Considering the person variable, our study has pointed to stronger associations between injuries sustained during vehicle boarding and alighting and (a) older adults, (b) men, and (c) persons of non-White or Black race. These demographic factors lend insight into more at-risk persons. The impact of safe vehicle access is certainly influenced by the context in which one lives and, thereby, the societal expectations and contextual necessity of safe vehicle access. The environment, in this case, considers the source of the dataset, the United States, where the majority of residents continue to own personal vehicles as their means of community transportation. When compared across countries in a multi-nation survey by Statistica (United States, China, Germany, the United Kingdom, Brazil, and South Korea), the United States had the highest percentage of vehicle ownership at 75% (Armstrong, 2022). This highlights the general trend within the context of our investigation—a society that continues to primarily practice autonomy in community transportation throughout day-to-day life. In sum, through the lens of the PEO model, the number of boarding and alighting injuries begs continued attention toward safe vehicle entry and exit.

A safe vehicle entry method encompasses sitting while facing away from the vehicle and then, once seated, bringing feet over the threshold and turning to face the front of the vehicle (Ignite Healthwise, LLC Staff, 2024). This approach ensures that an individual has either both feet on the ground or is seated and is upright at all points (e.g., never lifting one leg or leaning to duck under the vehicle doorway). A second option focuses on maintaining three points of contact at all times until seated—either one hand on the car as two feet remain on the ground, or two hands on the car (perhaps one on the door and the other on the door frame) while lifting one leg to enter the vehicle (Safe at Work California, 2020). Although these methods are taught in more direct one-on-one settings (e.g., taught by a physical or occupational therapist prior to discharge from a rehabilitation facility), there may be opportunities for preventative community education on vehicle transfer safety. A recent systematic review of fall injury prevention education reports that these education interventions were effective in increasing fall prevention behaviors among community-dwelling older adults (Ong et al., 2021). Similarly, safe vehicle entry and exit education could be disseminated through community and senior centers, as a component of a community health fair, or asynchronously through prerecorded education on older adult-facing websites and social media. In addition, vehicle designers might continue to consider incorporating universal design approaches to ensure vehicle safety at all points of use—from entry to exit.

### Limitations

Our study was modeled after an existing study by Dellinger, Boyd, and Haileyesus (2008), utilizing the same data source. However, some data described in their original article was not available in the current dataset. Therefore, the present report does not provide sub-analyses on boarding and alighting injuries specific to fall-related injuries. Additionally, without medical note data, additional investigation into external (contextual) and person-related (comorbidities) risk factors could not be assessed. Future work should focus on expanding access to de-identified medical note data that will shed light on key points of risk. Geographic region and spread of the NEISS-AIP data are not reported in the data set, and, therefore, the generalizability of these findings cannot be confirmed. There may be an opportunity to address this limitation in future research if the NEISS-AIP data set can be linked to other data sets that provide additional demographic data points. An example of this is the LINKAGE approach to the Health and Retirement Study (HMS) data set. Specifically, the HMS data set can be linked to Medicare Part C data to expand the variable list with which researchers can investigate topics of interest. Finally, there is a paucity in the literature on vehicle entry- and exit-related injury prevalence, outcomes, or prevention strategies. Therefore, our study’s foundation is limited to emerging literature. Future research should continue to expand this area of study.

## Conclusions

In sum, this study extends insight into the prevalence and profile of boarding and alighting injuries among a representative American sample. The annualized estimate of injury is 209,797 with a higher rate of injury among older adults. As compared to earlier estimates, the hospitalization rate for older adults is over 10 times higher for those ≥65 years. Future research should continue to prospectively investigate risk and mitigation factors.
